# Towards Rehabilitation Robotics: Off-the-Shelf BCI Control of Anthropomorphic Robotic Arms

**DOI:** 10.1155/2017/5708937

**Published:** 2017-08-29

**Authors:** Alkinoos Athanasiou, Ioannis Xygonakis, Niki Pandria, Panagiotis Kartsidis, George Arfaras, Kyriaki Rafailia Kavazidi, Nicolas Foroglou, Alexander Astaras, Panagiotis D. Bamidis

**Affiliations:** ^1^Biomedical Electronics Robotics & Devices (BERD) Group, Lab of Medical Physics, Faculty of Medicine, School of Health Sciences, Aristotle University of Thessaloniki (AUTH), 54124 Thessaloniki, Greece; ^2^1st Department of Neurosurgery, “AHEPA” University General Hospital, Aristotle University of Thessaloniki (AUTH), 54636 Thessaloniki, Greece; ^3^Robotics Laboratory, Computer Science Department, American College of Thessaloniki (ACT), 55535 Thessaloniki, Greece

## Abstract

Advances in neural interfaces have demonstrated remarkable results in the direction of replacing and restoring lost sensorimotor function in human patients. Noninvasive brain-computer interfaces (BCIs) are popular due to considerable advantages including simplicity, safety, and low cost, while recent advances aim at improving past technological and neurophysiological limitations. Taking into account the neurophysiological alterations of disabled individuals, investigating brain connectivity features for implementation of BCI control holds special importance. Off-the-shelf BCI systems are based on fast, reproducible detection of mental activity and can be implemented in neurorobotic applications. Moreover, social Human-Robot Interaction (HRI) is increasingly important in rehabilitation robotics development. In this paper, we present our progress and goals towards developing off-the-shelf BCI-controlled anthropomorphic robotic arms for assistive technologies and rehabilitation applications. We account for robotics development, BCI implementation, and qualitative assessment of HRI characteristics of the system. Furthermore, we present two illustrative experimental applications of the BCI-controlled arms, a study of motor imagery modalities on healthy individuals' BCI performance, and a pilot investigation on spinal cord injured patients' BCI control and brain connectivity. We discuss strengths and limitations of our design and propose further steps on development and neurophysiological study, including implementation of connectivity features as BCI modality.

## 1. Introduction

Advances in neural interfaces including implantable neural prosthetics and brain-computer interfaces (BCIs) have recently demonstrated remarkable results in the direction of replacing [1, 2] or even restoring [3, 4] long-lost sensorimotor function in human patients. Pathological conditions like spinal cord injury (SCI), amyotrophic lateral sclerosis, and stroke, among others, compromise an individual's physical and psychological well-being and result in social seclusion due to the severance between volition and the ability to act [5]. SCI in particular results in disconnection of afferent and efferent neural pathways and can cause permanent sensorimotor disability, often without any cognitive alteration, which negatively impacts the lives of the victims and their families [6]. BCIs aim to bridge this disconnection by detecting and decoding brain activity, thus allowing patients to control external devices, robotics, and exoskeletons [1–5]. Nonetheless, chronic SCI has been demonstrated to induce neurophysiological changes in brain structure [7] and function, both at resting state [8] and during sensorimotor process [9]. These neurophysiological changes could negatively affect the design and development of robust and durable BCIs for motor restoration [4, 10]; hence they should be systematically investigated further [11].

Despite recent technological breakthroughs in BCI research, in terms of reliability, accuracy, and speed, the best results in robotics and neural prosthesis control have been demonstrated by invasive technology (neural implants) [1, 2, 12, 13]. Noninvasive BCIs, on the other hand, are far more widespread and hold many relative advantages, including simplicity, safety, lower cost, and range of applications [14, 15]. Moreover, novel paradigms and recent advances in noninvasive BCI protocols also aim at progressively improving past technological and neurophysiological limitations to levels comparable to invasive BCIs [16, 17]. Such a paradigm, taking into consideration the aforementioned neurophysiological alterations that disabled individuals demonstrate compared to healthy users [11], lies with investigating brain connectivity features for implementation of BCI control [17–19]. Commercial electroencephalography (EEG) BCI systems, as another approach, are based on fast, reproducible detection of a low number of mental states and have taken the spotlight in consumer applications. They are even increasingly considered for robotics control [20, 21], often employing the detection of motor imagery (MI) states. The mental execution of an action, MI, displays similarities in brain activation [22, 23] with physical execution and as such has also been deployed in rehabilitation and BCI applications for disabled individuals [5, 14]. MI consists of a visual and a kinesthetic component, corresponding to two task-dependent and distinct neural contributing systems [24–26]. Visual motor imagery (VMI) implies that a representation of the motor task is provided (e.g., video or avatar), while kinesthetic motor imagery (KMI) is based on internal simulation or rehearsal of the task. While networks formed during VMI and KMI both involve motor related cortical areas, VMI also involves the occipital and superior parietal cortical areas while KMI involves the inferior parietal cortex [24, 26].

Even past the challenges and limitations of BCI systems, the design of a robotic arm for medical engineering applications, such as rehabilitation and assistive technologies for disabled individuals, constitutes a challenge on multiple fronts, including engineering problems, design requirements, and budget cost issues [27]. Designing a custom-made robotic arm allows for greater flexibility and negates the need to purchase expensive research-level robotics. It also raises several issues: reduced accessibility to directly comparable experimental findings by other research groups [28], lack of standardization, harder validation of experimental results, and increased difficulty in assessing suitability to nonspecific applications [29] compared to similar commercially available robotic products.

While programmable automation design can be traced back to Ancient Greece [30], modern transistor-based electronics during the latter half of the 20th century have allowed for complex electromechanical devices (mechatronics) of unprecedented programmability, precision, speed, strength, and durability. Subsequent integration of sensors and powerful digital microprocessors has increased the versatility of modern robots and medical applications (surgical applications, mechatronic prosthesis, and rehabilitation) are developing fast. Currently robotic systems are constantly under direct human control, but semiautonomous algorithms are also under development [31]. Constant advances in artificial intelligence algorithms mean that robots with medical decision support capabilities may be a likely next technological step [32]; however careful planning and public debate are required to ensure a human operator remains in the loop at all times to assume legal and ethical responsibility [33].

To that end, social robotics and Human-Robot Interaction (HRI) are considered important—yet sometimes overlooked—aspects of robotics development [34, 35]. User perception, satisfaction, and overall experience are of equal importance to hardware/software performance and quality standards [36]. Especially in fields such as rehabilitation that depends on human psychology, the success of a robot cannot be meaningfully assessed using technological performance and industrial integration criteria alone [36]. The accommodation of registering an external machine as a part of one's own body schema, which significantly affects the rehabilitation process, should also be taken into account [37]. HRI psychological and social characteristics can be investigated with questionnaires, carefully correlating psychological perception measurements with the characteristics of the robotic system used [34, 37]. The Godspeed questionnaire was selected for our purposes due to providing reproducible and comparable subjective measurements and sufficient coverage of HRI-related psychological states [36]. Such tools can prove invaluable in developing improved medical robotics particularly for prosthesis and rehabilitation applications [38].

In our previous work we have already presented the conceptual design and development of the Mercury robotic arm for biomedical applications and dealt with construction standards and validation tests [21]. We implemented a Body-Machine Interface (BMI) control module and conducted a pilot end-user assessment experimental study [39], focusing on both the technical characteristics and performance, as well as on HRI [36]. Our research team has since further improved the robotic arms in terms of anthropomorphism and allowing for movement along more Degrees-of-Freedom (DoFs) through the addition of a gripper resembling a human hand. We also improved the electronics and integrated a second symmetric Mercury arm into the system [40].

In the remainder of this paper we present our progress and goals towards developing off-the-shelf BCI-controlled robotic arms for assistive technologies and rehabilitation applications. In Materials and Methods, we first account for further development of the robotic arms and electronics, including a qualitative assessment study of the BMI module. We subsequently report on the implementation of the BCI control module using an off-the-shelf EEG-BCI system and the development of BCI-robotics communication; then we present two illustrative experimental applications of the BCI-controlled robotic arms. The first experiment is a study on healthy individuals to compare MI modalities for optimal BCI performance. The second experiment regards a comparative pilot investigation on SCI patients and healthy individuals for noninvasive control of multiple robotic arm motions and functional connectivity [41]. In Results and Discussion we first report the results of these two illustrative experiments regarding training, performance, and qualitative assessment, as well as briefly presenting pilot findings regarding brain networks. We then discuss the strengths and limitations of our experimental design and propose further steps on robotic development and neurophysiological study.

## 2. Materials and Methods

### 2.1. Mercury: Short Account on Development Milestones of the Robotics

#### 2.1.1. The Robotic Arms Platform

The Mercury robotic arm system has been developed as a customized design by our team for two technological generations so far [39, 40]. Design requirements focused on biomedical engineering applications, specifically intuitive remote robotic control, HRI research, and medical robotics for rehabilitation. Emphasis was placed primarily on fluid, anthropomorphic motion, fast response times to control triggers, and low fabrication cost. At a lower priority we regarded precision of movement and heavy lifting capability. Since the development of the robotic system has been presented elsewhere [39, 40], hereby we briefly report the technological characteristics of the system used in our current experimentation.

**Figure 1:**
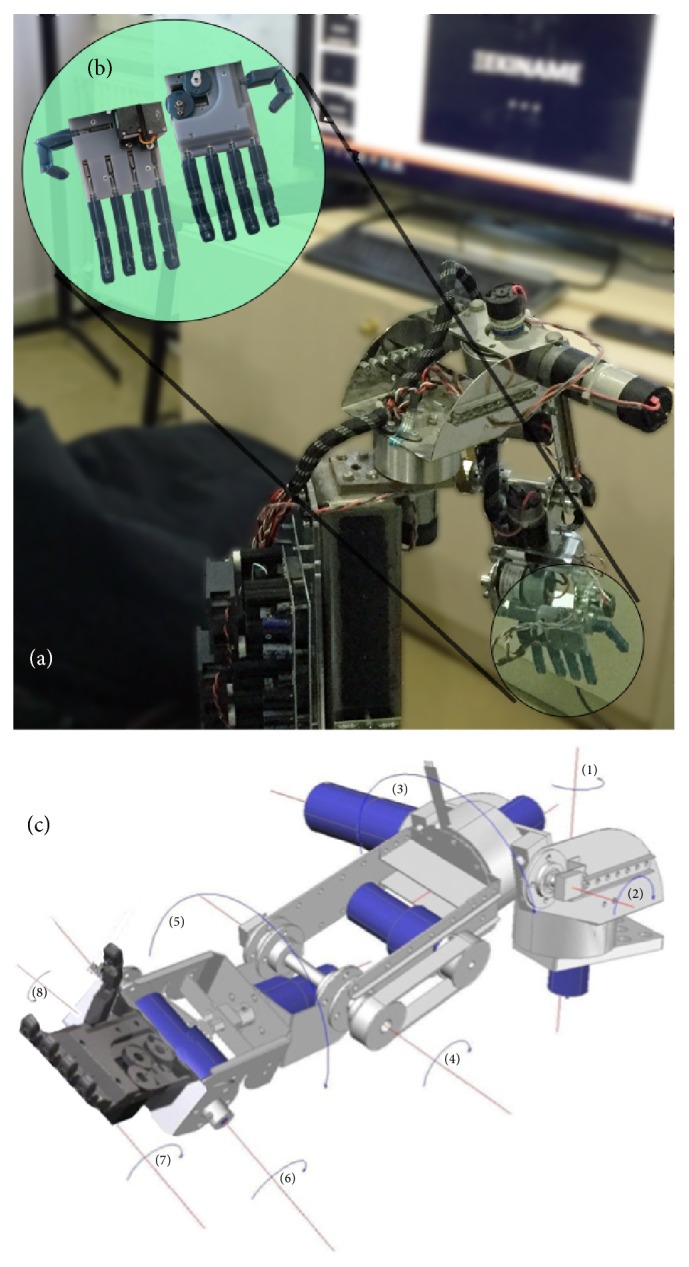
Current generation of Mercury robotic arm: (a) the robotic arm in position during an illustrative experiment, (b) the 3D-printed gripper (in focus circle), and (c) schematic of the 8 DoFs of the robotic arm. Mercury arms are house-built, of low cost, and anthropomorphic.

The Mercury robotic platform comprises a robotic arm currently capable of movement along 8 DoFs (at shoulder, elbow, wrist, hand gripper, and thumb joints), as well as a choice between two control modules [42]: (a) a custom-designed BMI capable of sensing the movement of a human operator's arm and (b) a commercially available BCI (EPOC, Emotiv, USA) which was integrated into the system. The system uses commercially available DC motors to provide movement: (a) along Cartesian vectors for the robotic shoulder joint (2 DoFs: “right-left” and “up-down”), the elbow joint (1 DoF: “up-down”), and the wrist joint (1 DoF: “up-down”) and (b) 2 DoFs along rotation axis between the “shoulder/elbow” and “elbow/wrist” parts. Two servomotors complement the robotic arm's movement capabilities, allowing for gripping small objects with a 3D printed, anthropomorphic gripper: 1 DoF is used for the thumb and 1 DoF for the rest of the fingers (Figure 1).

#### 2.1.2. The Body-Machine Interface

The BMI control module for the Mercury robotic arm has been described in previous work in terms of design, construction, cost, and features [39]. In the current section we provide a synopsis of the BMI module simply to facilitate comprehension of the technological evolution of our overall experimental robotic setup. The Mercury BMI comprises an exoskeletal position sensing harness (EPSN), which is worn by the user around their arm. It uses analogue resistance sensors to capture the movements of the shoulder, elbow, and wrist, as well as the gripping movement of the human hand. Movement is captured along 6 DoFs, a subset of the actual capabilities of the real human arm but enough to provide a realistic reproduction of the movement of the aforementioned joints.

During the design process of the EPSN, emphasis was placed on rapid capture and transfer of control signals to the Mercury robotic arm, allowing it to replicate the movement of the human operator's arm in a fluid, anthropomorphic fashion. For this purpose analogue classical automation control circuits were used to calculate analogue control signals subsequently fed to an Atmel ATmega2560 microprocessor. The microprocessor handled digitization, interface to a PC, and generation of the control signals for the Mercury robotic arm. Initial experiments using the EPSN focused on HRI, specifically the time required for first-time human operators to develop the skills to control the Mercury robotic arm and perform basic tasks such as knocking, gripping, lifting, and placing small objects [39]. Those initial experiments also gathered perceived psychometric characteristics from pilot testers, classified by age, sex, level of education, and familiarization with electronics and robotics technology [43, 44]. These comparisons revealed not only a tendency of female pilot testers and those unfamiliar with robotics to perceive the Mercury robotic arm as being more humanlike, but also a disillusionment effect being induced to all participants after the pilot testing.

### 2.2. Brain-Computer Interface Module Development

#### 2.2.1. Off-the-Shelf Brain-Computer Interface

Advances in both hardware and software technologies rendered real-time EEG processing a possibility, including detection and identification of brain activity features for use in BCIs. Currently there are several BCI systems available commercially, one of which is the Emotiv EPOC (USA), sold around $300, which is significantly lower than most medical EEG devices. It is a portable, wireless EEG recording device that has 14 dry electrodes arranged according to the international 10–20 System and can be easily mounted to the user's head. The device operates at an internal sampling rate of 2048 Hz and the data are transmitted wirelessly at 2.4 GHz to a USB dongle with a sampling rate of 128 Hz. The BCI capabilities of the device are accessed by the Cognitiv suite and rely on Event Related Desynchronization (ERD). The user initially needs to record a resting state EEG after which he is able to train up to four mental commands, using a machine-learning pipeline to teach the BCI how he visualizes. The pipeline operates along the stages of preprocessing, feature extraction, reduction of dimensionality, and classifier training. Following the training, the suite will continually attempt to identify the trained commands by analyzing the user's EEG. During this process, the suite presents a floating box that will execute any mental command that it identifies, and the action power, corresponding to the level of confidence of each classification.

#### 2.2.2. Communication between BCI and Robotics

**Figure 2:**
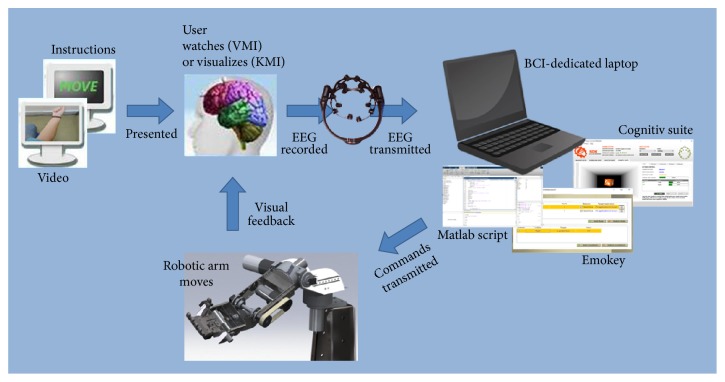
Schematic of Brain-Computer Interface loop: using off-the-shelf EEG-BCI for control of house-built robotic arms.

In order to achieve online communication between the commercial BCI application and the robotic arms, the trained BCI classes are mapped in real-time to computer controls, using a combination of the BCI's native Emokey application (Emotiv, USA) and an in-house script, developed in Matlab environment (Mathworks, USA). In our implementation, the BCI is trained in only three classes: one for resting state and two for general “left” or “right” directions, using either visual or kinesthetic motor imagery. Each BCI class is linked to a specific key button, which is enabled when the detected mental state corresponds to that class. Then the script accepts the corresponding command as input and transmits it through a serial port, with Baud Rate 9600, to the on-board microcontroller unit for each Mercury robotic arm (Figure 2). The arms' units translate that input to specific positional coordinates for each of the 8 DoFs' motor. Using this approach, we achieve a move reaction time of the system that approximates 0.2 seconds.

### 2.3. Current Experimental Setup

The Bioethics & Ethics Committee of Faculty of Medicine, Aristotle University of Thessaloniki, approved the experimental protocol. All experiments were conducted after the participants providing informed consent and no remuneration was given. To facilitate the integration of the robotic arms (or the limb presentation during EEG recording) into the participants' own body schema, their arms and body were covered with a black curtain [37] during all experimental procedures. Wherever visual cues were used (video of arms or legs moving) the presented limbs were always matched with regard to the participant's sex. Furthermore, none of the participants in any experiment reported prior experience with MI practices or BCI experiments (characterized as BCI-naïve [45]). Finally, the participants reported on their user experience, rating the HRI characteristics of the system by answering the Godspeed questionnaire [36], translated in the Greek language [44].

**Figure 3:**
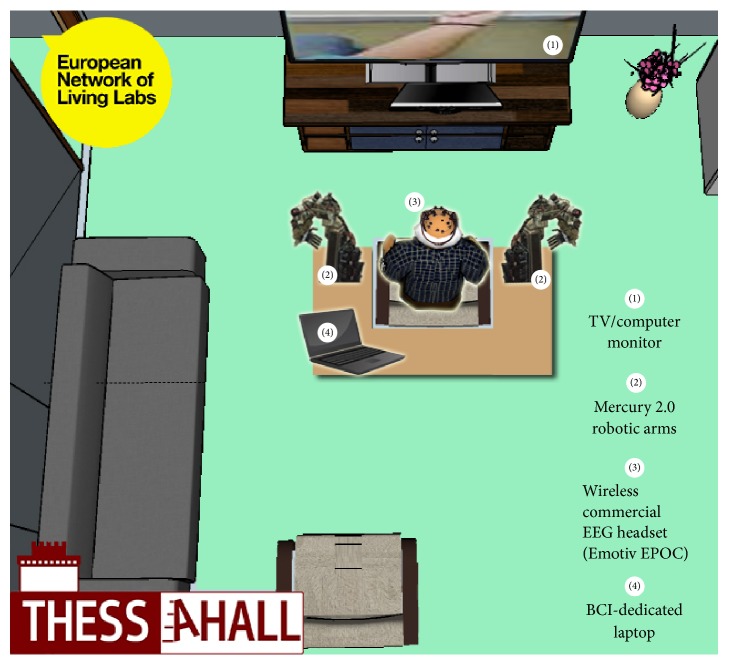
Overview of the experimental setup in the Thess-AHALL Living Lab. The figure is modified with authors' permission [46].

All experimental parts that involved the use of the robotic arms were conducted in the Thessaloniki Active and Healthy Ageing Living Lab technology showcase room (Thess-AHALL, member of ENoLL, http://www.aha-livinglabs.com, http://medphys.med.auth.gr) [46, 47] that is equipped with accelerometers for fall detection and observation cameras [48]. Participants comfortably sat on a chair, while disabled individuals sat on a wheelchair, docked between the two robotic arms and facing a 42′′ TV/computer monitor located a meter away (Figure 3). EEG recordings were taken from an Emotiv EPOC headset with a sampling rate of 128 Hz and wirelessly transmitted to the BCI-dedicated laptop that was mounted on the frame and operated by the investigator, situated behind the participant.

The experimental parts that involved the use of high-resolution EEG recording were conducted in a specially designed magnetic shielded room for recordings with presentation capabilities and audiovisual monitoring. The participants sat on an inclined armchair inside the room, while facing a 21′′ computer monitor located a meter away. Recordings were obtained using the 10-5 international electrode system for high-resolution EEG [49] with a sampling rate of 1000 Hz and impedance threshold set at 10 kOhm. An active electrodes cap was used (Brain Products, Germany) connected to a 128-channel EEG (Nihon-Kohden, Japan).

### 2.4. Qualitative Assessment Experiment: Comparison of MI Modalities

The first of the two illustrative experimental applications was a qualitative assessment study, comparing MI modalities for control of the BCI-controlled robotic arms by healthy individuals with regard to BCI training and optimal performance [50]. The participants were trained to use visual and kinesthetic cues to control simple motor tasks of the two robotic arms and we assessed their skill training and success rates.

#### 2.4.1. Subjects and Training Procedure

In total thirty healthy participants were included in the study, 18 male (60%) and 12 female (40%), ranging from 19 to 46 years (median age 24 years). All 12 female and 14 of the male participants declared that they were right-handed. From the rest of the male participants, 2 declared being left-handed and 2 being ambidextrous.

**Figure 4:**
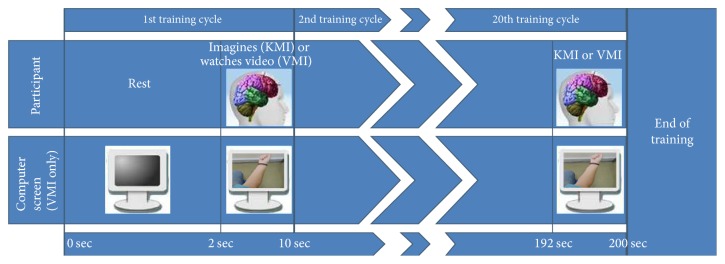
The training procedure of the qualitative assessment experiment.

Kinesthetic motor imagery (KMI) modality was trained first. The participants were asked to relax and resting state EEG with eyes-open was first trained as the neutral BCI class. All participants were then asked to strongly imagine a commonly performed (daily routine) movement for each hand (left and right) without actually moving their limbs. A “left” and “right” BCI class was trained accordingly, 20 times each. Always the “left” class was trained first and training was conducted in blocks of five training cycles. Each cycle consisted of 8 seconds of continuous recording of the mental state (“training”) and 2 seconds of rest (Figure 4), while the achieved training skill percentage and the action power of each cycle were recorded (action power threshold was set at 50%). When the KMI training was concluded (Figure 4), the participants rested for 2 minutes before attempting to control the robotic arms (control trials are described in Section 2.4.2).

The participants rested for 10 minutes after the KMI control trials and then the VMI modality was trained. The training procedure was the same but instead of imagining a movement, during the “training” cycle, a video played on the TV monitor (left or right forearm pronation). Again when the VMI training was concluded, 2-minute rest intervened before the participants attempted to control the robotic arms.

#### 2.4.2. Robotic Arm Control Trials and Success Rates

The participants attempted to control the “elbow/wrist” rotational DoF of each robotic arm. First they attempted to move the right robotic arm 10 times with the “right” BCI class and then the left robotic arm 10 times with the “left” BCI class. Each trial cycle lasted 10 seconds with a 2-second rest between them and a successful trial was marked by any detection of the correct BCI class during the 10-second period. When the correct class was detected the relevant DoF moved (corresponding to pronation), while it remained idle otherwise.

**Figure 5:**
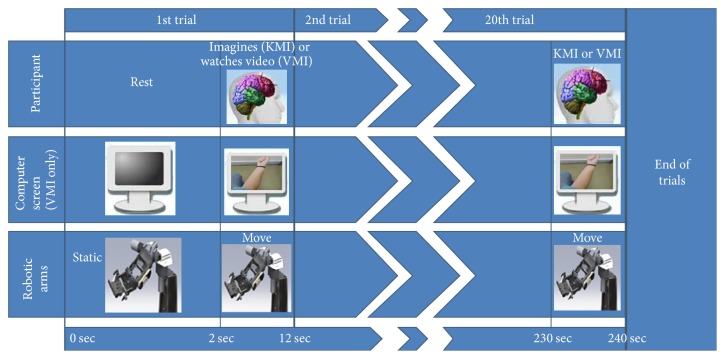
Overview of robotic arm control trials during the qualitative assessment experiment.

For the control trials using KMI, only a command was given to the participants to attempt to control the robotic during the trial cycle. For the control trials using VMI, during the trial cycle, on the TV monitor the same video that the participants were trained to played and no command was given (Figure 5). Success rate was recorded for each robotic arm and imagery modality (successful trials in 10 consecutive trial cycles of right or left robotic arm control in either KMI or VMI). Success rates for each imagery modality were also calculated (successful trials in 20 trial cycles of both robotic arms control).

#### 2.4.3. Statistical Analysis

*(1) Demographics. *Six participants who did not succeed in passing action power threshold during skill training (50%) were excluded from further analysis. All further comparisons regarding demographics (as well as skill training, success scores, and Godspeed questionnaire, as presented in next sections) were made on the remaining participants (*n* = 24). Planned comparisons explored the age differences across the remaining participants using as grouping factor the gender/sex (female, male). The age was tested for normality following Shapiro-Wilk Test [51, 52] after controlling for sex. However, age did not meet the normality assumption when controlled for sex. Therefore, age differences between sexes were explored using Mann–Whitney (*U*) Test. We did not control for hand dominance as grouping factor because the majority of the remaining participants were right-handed. Significant age differences between female and male participants were not found (*U* = 68; *p* = 0.816).

*(2) Kinesthetic against Visual Motor Imagery Skill Training. *KMI skill training scores were compared against VMI skill training scores (a) for all remaining participants and (b) for participants grouped by gender. For all participants, scores were compared for both hands (left and right hand separately) and also across training blocks after those being tested for normality assumption. The differences between Kinesthetic and Visual Skill scores were normally distributed across training blocks for both hands. Therefore, Paired *t*-tests were planned for each training block and for both hands separately. After grouping by gender, we compared again KMI and VMI skill training scores across training blocks and for both hands separately. For the aforementioned statistical analyses Paired *t*-tests were used since differences (Kinesthetic-Visual Skill scores) were still normally distributed after controlling for sex.

*(3) Kinesthetic against Visual Motor Imagery Success Scores in Robotic Arm Control*. Planned comparisons regarding the KMI and VMI success scores of BCI robotic arms (both right and left) control were performed. The number of successful trials in ten consecutive trials was defined as success scores. Statistical analysis was performed using Wilcoxon Signed Ranks test as KMI and VMI success scores were obtained by the same participant.

*(4) Godspeed*. Godspeed scores of each key concept (Anthropomorphism, Animosity, Likeability, Perceived Intelligence, and total Godspeed) score were analyzed as interval variables (for more information see Section 2.6.). Therefore, we tested for normality assumption grouping by sex (female, male) and used Shapiro-Wilk Test. Likeability, Perceived Intelligence, Perceived Safety, and total Godspeed score were found to be normally distributed and *t*-tests were performed between female and male participants. Anthropomorphism and Animosity were analyzed between two groups following Mann–Whitney (*U*) test.

### 2.5. Pilot Patient Investigation: BCI Control and Functional Brain Connectivity

The second of the two illustrative experimental applications is an ongoing pilot study that involves SCI patients and healthy individuals controlling multiple DoFs of the robotic arms, as well as an investigation of their brain connectivity [53]. The participants are trained with visual cues of various arm movements or walking and then use kinesthetic cues for BCI control of the robotic arms. Apart from assessing their performance, we moreover perform a pilot analysis of the functional brain networks formed for each different movement.

Three SCI patients were already recruited for participation in the pilot study, one female (28 years old) and two male (52 and 47 years old), as well as three age and sex matched healthy individuals as control group. The patients' neurological level of injury was C4, C4, and T7, respectively and their Asia Impairment Scale classification was D, C (incomplete injuries), and A (complete injury), respectively. The protocol involves a full neurological examination using the International Standards for Classification of Spinal Cord Injury [54] and assessment of their functional status using the Spinal Cord Independence Measure III [55] in the Greek language (g-SCIM-III) [56]. Moreover, the protocol also involves healthy and patient participants both answering Vividness of Visual Imagery Questionnaire (VVIQ) [57], Beck Depression Inventory (BDI) [58, 59], and Rosenberg Self-esteem Questionnaire (RSQ) [60, 61]. Since the investigation is ongoing and more patients are expected to be recruited, our focus hereby will be on presenting an overview of the methodological aspects of the study, as well as provisional results regarding functional connectivity from one subject and healthy control.

#### 2.5.1. High-Resolution EEG Recording during Multiple Movements

**Figure 6:**
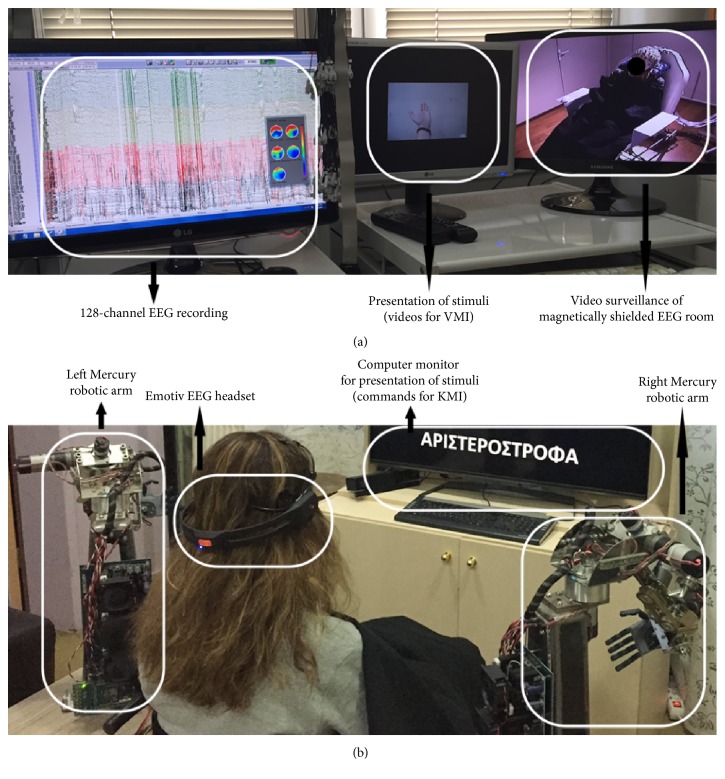
28-year-old female SCI patient participating in the pilot investigation: (a) 1st part of the experiment, 128-channel EEG recording during oddball presentation of multiple limb movements (visual imagery); (b) 2nd part of the experiment, control of robotic arms using a commercial EEG-BCI headset, employing mental rehearsal of movements (kinesthetic imagery).

While under high-resolution EEG recording (as described in Section 2.3) the participants watched random video recordings of upper limbs performing movements of all DoFs or lower limbs walking. The participants attempted to register these movements as being their own [37], without moving their own limbs (VMI) (Figure 6). The presentation followed an oddball paradigm, displaying randomly 9 repetitions of 34 videos, divided into 3 sets with 10-minute rest between them. For each of 8 possible DoFs of the arms, both directions of movement were displayed, for both left and right arm, totaling 32 videos of upper limbs. The remaining 2 videos were walking (from walker's perspective) and an oddball wildlife video. All videos had duration of 5 seconds, followed by 4 seconds of black screen.

#### 2.5.2. BCI Control of Robotic Arms

In the second part of the experiment, the participants used the commercial EEG-BCI to control the robotic arms. Three BCI classes were trained: resting state, left, and right. The participants were asked to visualize the videos they were presented during the previous part during the training of left and right. Each direction was trained 20 times, each cycle lasting 8 seconds, followed by 2 seconds of rest.

After the system's training to each participant's brainwaves, they were asked to follow presented instructions, corresponding to specific DoFs of the robotic arms and to specific direction of movement. The participants attempted to visualize the same movements to achieve control (KMI), without moving their limbs, while the BCI detected one of the three aforementioned classes. The presentation followed a pseudorandom routine that included an instruction to perform each of 32 possible arm movements once. Each instruction lasted 30 seconds, followed by 5 seconds of rest period. The participants' performance in each movement was rated on a 0–5 scale and an overall percentage score was calculated to denote overall BCI performance.

#### 2.5.3. Signal Analysis and Brain Networks

*(1) Preprocessing*. The acquired high-resolution raw EEG signals were band-pass filtered between 2 and 50 Hz using a zero-phase finite impulse response filter, downsampled at 100 Hz, and rereferenced to the common average reference (CAR) [62]. Triggers were set at the onset of each visual stimulus, using the signal from an optic diode, and epochs were extracted from −2000 msec prestimulus to 4000 poststimulus. Epochs were visually inspected and the heavily artifactual contaminated ones (due to subject movements, spasticity, and electrode disconnection) were rejected. The remaining epochs were averaged according to the event type (motor imagery of different movements), resulting in 34 average epochs per subject.

*(2) Cortical Current Density Estimation*. In order to improve spatial resolution of the data and counter smearing caused by the volume conduction effect we deployed cortical current density estimation (CCD) [62] using the Brainstorm [63] toolbox for Matlab. CCD essentially maps the sensor potentials to dipole current distribution that are assumed in fixed positions over the cortex. Dipoles also are referred to as sources, model electrical activity of neuronal groups that fire synchronously [64]. CCD requires first a model of the head conveying information about the electrical properties and geometry of different parts of the head (e.g., scalp, skull, and cortex), electrode position, and source space dipole positions [65]. The Montreal Neurological Institute (MNI) COLIN 27 MRI [66, 67] was used as default subject anatomy to compute a three-shell (scalp, skull, and cortex) head model with boundary element method (BEM) using OpenMEEG [68] via Brainstorm. The cortical surface is assumed as source space. Having the head model and sensor data, CCD estimation was performed using standardized LORETA (sLORETA) method [69] with dipole orientation (5023) constrained normally to the cortex [70]. Noise covariance matrix was estimated on resting state data that take place at the start of each session and was regularized.

**Figure 7:**
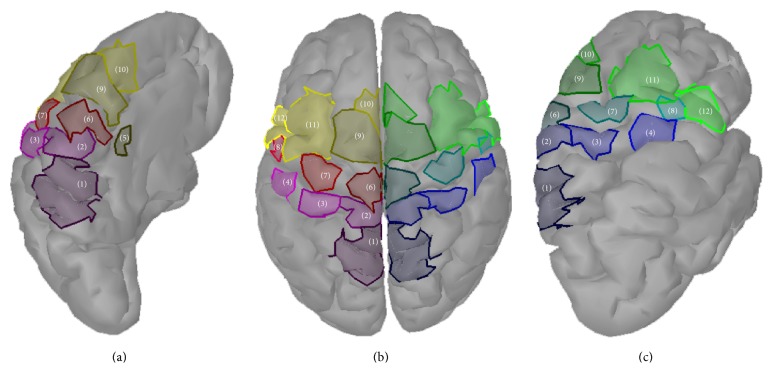
Regions of interest (ROIs) for connectivity analysis at the cortical level: (a) midline surface, left hemisphere, (b) top view, both hemispheres, and (c) lateral view, right hemisphere. (1): SAC, (2): S1F, (3): S1H, (4): S2, (5): CMA, (6): M1F, (7): M1H, (8): M1L, (9): SMA, (10): pSMA, (11): PMd, and (12): PMv.

*(3) Functional Connectivity*. After solving the inverse problem of the average trials functional connectivity was performed on the source domain, analyzing the connectivity between 24 cortical regions of interest (ROIs), 12 in each hemisphere (Figure 7): Somatosensory Association Cortex (SAC), Primary Foot Somatosensory Area (S1F), Primary Hand Somatosensory Area (S1H), Secondary Somatosensory Area (S2), Cingulate Motor Area (CMA), Primary Foot Motor Area (M1F), Primary Hand Motor Area (M1H), Primary Lip Motor Area (M1L), Supplementary Motor Area (SMA), pre-Supplementary Motor Area (pSMA), Dorsal Premotor Cortex (PMd), and Ventral Premotor Cortex (PMv). Scouts were defined as ROIs, in the same manner as in our previous study based on neuroanatomical landmarks and Brodmann areas [71], locating scouts on the MNI cortical surface. Connectivity between those areas was calculated for the time period of −1000 msec prestimulus to 2000 msec poststimulus on each of the 34 averaged epochs, using Granger causality [72], for each subject. Networks were calculated for delta (1–4 Hz), theta (4–7 Hz), alpha (8–13 Hz), and beta (13–30 Hz) brainwave bands. Then the functional networks were comparatively assessed, displaying connections with power of at least 60% of the connection with highest power for each network.

### 2.6. Godspeed Questionnaire Translation and Statistical Manipulation

The Godspeed questionnaire consists of five semantic differential scales, equipped with Likert type scaling evaluating the attitude towards robots in the subcategories of Anthropomorphism, Animacy, Likeability, Perceived Intelligence, and Perceived Safety [36]. Our team performed a double-blind forward and backward translation and adaptation to the Greek language [44]. Accuracy of the procedure was evaluated by a third independent researcher and concepts that needed further resolution were pinpointed and put to the same procedure again, in order to produce an accurate adaptation. The Greek version of the questionnaire (Godspeed-g) was used both in the BMI validation study [43] and the current experimental applications and has also been made available through the original questionnaire's official webpage [73]. Despite the criticism that the original questionnaire has attracted in terms of redundancy and suitability [74], it remains the most widely applied tool in studying user perception of robots [75].

As with the original one, in the translated version, each semantic differential scale represents a key concept enclosing a short questionnaire. Each short questionnaire results in a score adding the ratings of the respondent. However, in the last two questions of Perceived Safety subcategory reversed rating was used, to associate the lower scores to the negative assessment, as is the case with the other items of the questionnaire [76]. Finally, a total Godspeed score could be calculated adding the scores of each key concept. Semantic differential data can be analyzed as any other rating data, as both Likert scales and semantic differential scales are rating scales and the distributions of the responses are not forced [77]. The analysis of Godspeed data was performed using the guidelines of H. N. J. Boone and D. A. Boone (2012) [78].

## 3. Results and Discussion

### 3.1. Qualitative Assessment Experiment

#### 3.1.1. Kinesthetic against Visual Motor Imagery Skill Training

Participants achieved higher median skill training percentage using KMI. That for the left arm was 26.5% (1st training block), 56.5% (2nd), 75.5% (3rd), and 72.5% (4th) for KMI and 20,5%, 54.5%, 71.5%, and 73%, respectively, for VMI. For the right arm it was 14.5%, 26%, 36%, and 24.5% for KMI and 8.5%, 17%, 17,5%, and 14.5% for VMI. There was a fatigue effect, median skill training percentage dropping from 3rd training block to 4th in all settings but left arm VMI.

Statistical testing resulted in significant difference between KMI and VMI skill training score only for the right hand extracted by training block 1 (*t*(23) = 2.151; *p* = 0.042) and block 2 (*t*(23) = 2.181; *p* = 0.040) indicating that KMI skill training scores are higher than those of VMI in training blocks 1 and 2. Statistically significant findings were found neither at training blocks 3 and 4 nor for the left hand across any training block. When discriminating participants by sex, marginally significant difference between KMI and VMI skill scores was found for female participants in training block 2 (*t*(11) = 2.136; *p* = 0.056) favoring KMI training against VMI. Male participants' scores between KMI and VMI training did not reach significance across training blocks. Following the same analysis for the left hand did not yield any significant outcome.

#### 3.1.2. Success Scores in Robotic Arm Control and Godspeed Questionnaire

**Figure 8:**
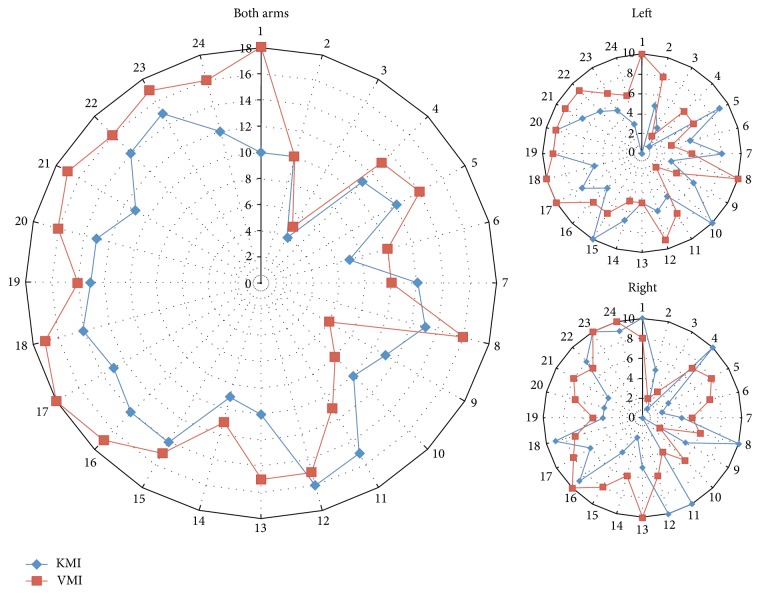
KMI against VMI success scores for 24 participants above action power threshold. Most participants performed better with VMI but the difference was not statistically significant.

Median success score was 7 for both left and right arm VMI, 5.5 for left arm KMI, and 5 for right arm KMI (Figure 8). Comparing success scores between KMI and VMI for right and left hand separately, the differences were not found statistically significant (right hand: *Z* = −0.945; *p* = 0.344; left hand: *Z* = −1.476; *p* = 0.140). Differences between female and male respondents to Godspeed questionnaire did not reach statistical significance (Anthropomorphism: *U* = 64, *p* = 0.643; Animosity: *U* = 70.5, *p* = 0.931; Likeability: *t*(16.226) = 0.483, *p* = 0.636; Perceived Intelligence: *t*(22) = 0.121, *p* = 0.905; Perceived Safety: *t*(22) = −0.861, *p* = 0.399) and total Godspeed score (*t*(22) = −0.085, *p* = 0.933).

#### 3.1.3. Discussion

While participants appeared to perform better using VMI rather than KMI as an imagery modality for BCI control, our analysis did not prove a statistically significant correlation [50]. Individual differences could play a role, since some participants performed better with KMI; it is worthwhile to explore this difference, as BCI control should be tailored to the needs of each individual [50]. Perception of the robot did not correlate to either performance or the sex of the operator. This qualitative assessment experiment provided us with important field insight on the operation of the robotic arms and the BCI control modality. Further comparisons, using this design, could include different users groups to perform either imagery type, in order to determine specific characteristics for each. Studying disabled users could also provide answers on the effect of neurological disability on imagery capacity and an ability to perform with BCI.

### 3.2. Pilot Patient Investigation

#### 3.2.1. Results and Discussion

**Figure 9:**
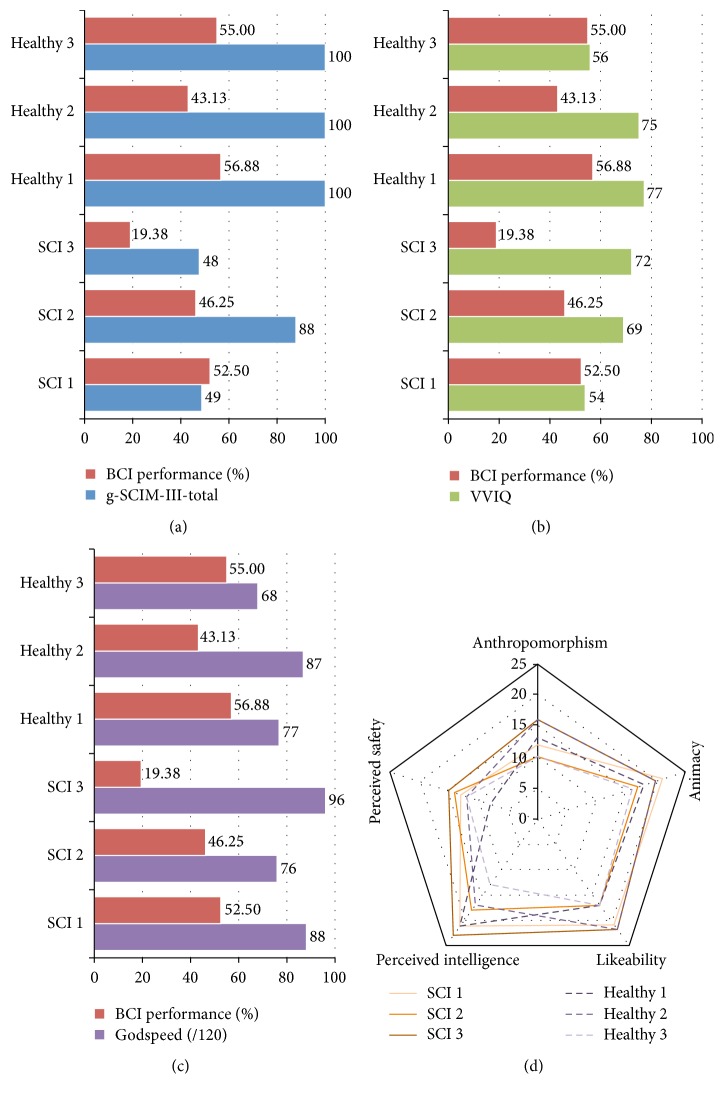
Performance in BCI control of patient and healthy participants in comparison to (a) their g-SCIM-III total score, (b) VVIQ score, and (c) Godspeed total score. Also (d) evaluation of the robotic arms in each separate Godspeed subcategory by participant.

Our experimental paradigm allows control of multiple DoFs of two robotic arms using a 3-class BCI implementation along with VMI training and the use of AI algorithms. As we have also shown in the proof-of-concept [53], disabled and healthy operators (Figure 9(a)) can achieve comparable, above-chance, performance levels in BCI control of the robotic arms (56.88%, 43.13%, and 55.00% by healthy participants and 52.00%, 46.25%, and 19.38% by SCI patients). While, after only a training session, for some movements only minimal control is achieved, further training sessions are suggested in order to improve performance. Nonetheless, in certain movements excellent performance was achieved (arms were moving towards the desired direction for the most part) and this finding was not correlated to intrinsic difficulty of any movement [53].

As this is an ongoing investigation and subject recruitment continues, we hereby only provisionally present results from connectivity analysis, while a comprehensive assessment of performance, psychometric evaluation, and functional connectivity will be performed with the conclusion of the study. Healthy participants scored 77, 75, and 56 (out of max 80) in the VVIQ questionnaire, while SCI patients scored 54, 69, and 72 (Figure 9(b)). Moreover, healthy participants evaluated the robotic arms with 77, 87, and 68 (out of max 120) in total Godspeed score and SCI patients gave 88, 76, and 96 (Figure 9(c)). The Godspeed subcategories whose scoring by healthy and SCI participants seems to differ are Perceived Safety and Perceived Intelligence (Figure 9(d)), although that is a trend that needs to be tested for statistical significance in data from more participants. In the categories of Anthropomorphism, Animacy, and Likeability, both groups gave almost identical answers (Figure 9(d)).

**Figure 10:**
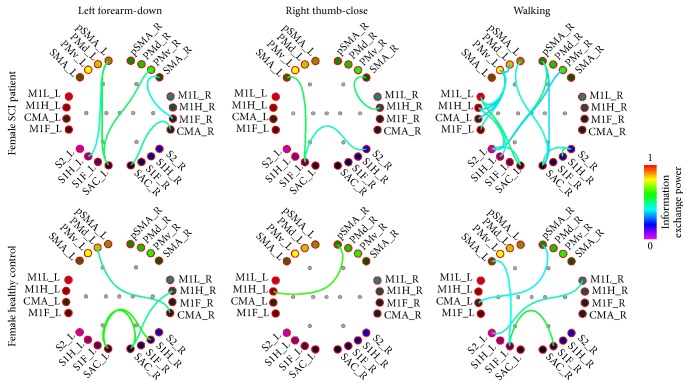
Functional connectivity networks formed in alpha brainwave band during different visual motor imagery tasks performed by an SCI patient and a sex and age matched healthy control participant (connections > 60% max power displayed).

In Figure 10, functional connectivity networks over the ROIs that we defined at the cortical level (seen in Figure 7) are presented for different motor tasks, performed by a female SCI patient and a healthy control.

Functional connectivity holds promise in classifying imagery of multiple classes (multiple different movements) or complex motions, based on imagery modalities. A possible automated approach would be to identify significant connections for each task using Network-Based Statistics (NBS). In our opinion, semiautonomous algorithms and AI should be part of a strategy to control multiple DoFs of robotic arms. Our BCI approach uses a 3-class implementation to achieve control of many (32 possible) DoFs but currently relies on research intervention. The low-class approach employed could be feasible both for BCI training and neurophysiological investigation. While training and functional connectivity study is performed using high-resolution EEG, it is highly impractical to use such systems for everyday BCI applications. Therefore, we aim to downscale the findings from high-resolution EEG regarding functional connectivity to control features for commercial low-resolution EEG-BCI headset. Moreover, other investigations could include trauma-induced brain reorganization with a focus on possible rehabilitation opportunities.

### 3.3. Future Steps

#### 3.3.1. Further Robotics Development

A natural milestone for future development is the integration of the BCI and robotic arms system into the operator's perceived body mental image [37]. From the user's point of view, this requires rapid, fluid, accurate, and predictable system performance. Furthermore, this necessity consequently corresponds to rapid processing of analogue BCI input: filtering and extraction of relevant brainwave information into the relevant robotic control signals in near-real-time (<100 ms). Maintaining time lag to a minimum is particularly important in order to avoid confusing the human brain's natural visual and tactile feedback loops. Determining the upper limit in response time lag is likely to depend on both the task and the user; we believe it will be meaningful to investigate this limit and the gradual deterioration of user control past it, across different types of tasks. Furthermore, we plan to investigate the operator's perception (from the HRI perspective) as response time lag varies across the aforementioned time limit.

Another important aspect of perceived body mental image that needs to be taken into account in further development is anthropomorphism. In our current technological generation, user perception of the robotic arm, as measured by Godspeed, did not correlate with performance [50]. As further robotics development would also focus on improving anthropomorphic characteristics of the system, as well as the users' perception, it would be interesting to identify possible correlations between advancements in that direction and performance, as well as to identify possible “uncanny valley”-like phenomena [74]. An important question, also with regard to real-time response of BCI-robotics systems, would be to investigate whether operators would expect more natural and fluid response from a near humanlike robotics system than from a more mechanical-looking one, and whether not meeting such expectations would affect either user perception or performance. Furthermore, as subject recruitment progresses through the ongoing study [53], we also aim to investigate correlations between operators' emotional state, perception of the robotics, and performance. Finally, further robotics development and associated experiments should focus on naturalistic scenarios and real-life applications, designed for both disabled and healthy end-users.

#### 3.3.2. Further Neurophysiological Investigation

There are several paradigms for sensorimotor BCI implementation that vary from machine learning to signal processing perspective. Current BCIs are capable of easily recognizing two classes, which translates to control of 1 DoF but usually fails to work with more classes. One of the biggest challenges of noninvasive motor imagery BCIs is the low spatial resolution of EEG, due to volume conduction effect [79]; hence spatial features extracted directly from EEG are poorly discriminative.

Cortical current density estimation methods can be deployed to compensate for the low spatial resolution of EEG, by reconstructing activation of cortical sources using EEG data and realistic head model, so essentially transforming sensor data to a higher dimension space, where spatial resolution is higher. Several studies concluded that features extracted from source space are superior over sensor based [70], and a recent published study has achieved sufficient discrimination of complex movements of the same limb, utilizing source imaging techniques [70].

One of the strongest requirements of Mercury BCI algorithm is natural control of a multi-DoF robotic arm, corresponding to multiclass in terms of decoding. Decoding brain activity is still an open challenge especially when it turns to multiple classes [80], although implementation of functional connectivity features for BCI class classification [71] is expected to provide applicable solutions [17]. A foreseeable future direction of BCI algorithms is to extract almost solely features from source space. Data driven ROI specification for each subject based on ICA could be used, instead of static ROIs for all subjects. Features extraction scheme will be based on a combination of spectral, spatial, and connectivity features to improve robustness. For classification, hierarchical approach seems appealing for the multiclass problem.

Such a processing pipeline is highly computational demanding, and at this stage of its development we work with offline analysis until the results are encouraging to proceed to real-time implementation. Recent advancements in Graphic Processing Units (GPUs) and Field-Programmable Gate Arrays (FPGAs), which have proven to be effective in rendering computationally demanding applications in real-time, could be employed for an online implementation of our paradigm.

High-resolution EEG data and its analysis for functional connectivity from multiple motor imageries are expected to provide insight in brain network adaptive and maladaptive reorganization that occurs after SCI [11]. Most published studies have focused on resting state connectivity and those that have used MI have not yet discriminated between different motor tasks. On the other hand, low-density EEG data recorded through KMI-BCI operation can be further studied for functional connectivity networks and compared to data gathered from high-resolution EEG recordings during VMI. Such an approach could possibly facilitate the downscaling of a network-based BCI in the future for multiple DoFs and control of complex movement sequences. This should also point towards whether this network classification is possible with affordable, off-the-shelf, noninvasive BCI devices and low-resolution EEG.

#### 3.3.3. Limitations

In the context of our design and experiments, we encountered several limitations. First of all, although demands for portability, ease of use, low cost, and availability made the selection of a commercial dry electrode EEG headset necessary, the accompanying commercial (and undisclosed) BCI algorithm did not meet the needs of our neurophysiological experimentation [21, 81]. Current BCI technology has not demonstrated autonomous control of multiple classes and this constitutes a challenging implementation that necessitates BCI algorithms tailored to the need of the specific task (multi-DoF control). Possible solutions could lie in the source space and connectivity-based BCIs [17]. The group's next steps include developing own, true online, algorithms to be tested for the control of the 8 DoFs of the robotic arms, making use of AI to support classification. Nonetheless, combining a commercial EEG headset with elaborate homemade BCI and real-time computational approaches to the source space is also a challenge to meet.

## 4. Conclusions

Advances in BCIs have demonstrated remarkable results in the direction of replacing and restoring lost sensorimotor function in human patients. Novel paradigms and recent advances in noninvasive BCI protocols aim at progressively improving past technological and neurophysiological limitations. Neurophysiological changes in the brain network level, induced by SCI, could prove critical in designing and developing robust and durable noninvasive BCIs for motor restoration and rehabilitation. Moreover, successful rehabilitation strategies should take into account user perception, satisfaction, and overall experience, alongside performance. We presented our implementation of BCI-controlled 8-DoF anthropomorphic robotic arms, using noninvasive off-the-shelf BCI technology. Moreover we presented two illustrative experimental applications on healthy individuals and SCI patients. Current, state-of-the-art, BCI technology is unable to control multiple DoFs but semiautonomous AI algorithms and connectivity-based BCIs could provide solutions towards that direction. Individual differences appear to play a role in motor imagery based BCIs and multiple training sessions are always encouraged in order to improve performance in robotic arm control. Functional connectivity holds promise in classifying imagery of multiple classes (multiple different movements) or complex motions, based on imagery modalities. Future development aims at facilitating the integration of BCI and robotic arm system into the operator's perceived body mental image, thus requiring rapid, fluid, accurate, predictable system performance and improved anthropomorphism. Online implementation of connectivity-based classifiers, although currently too computationally demanding, is expected to be soon feasible. High-resolution EEG data and its analysis for functional connectivity from multiple motor imageries are expected to provide insight in brain network adaptive and maladaptive reorganization that occurs after SCI and, subsequently, into promoting or preventing it accordingly [11].
